# The health and demographic impacts of the “Russian flu” pandemic in Switzerland in 1889/1890 and in the years thereafter

**DOI:** 10.1017/S0950268824001651

**Published:** 2024-11-19

**Authors:** Jocelyne Suter, Isabelle Devos, Katarina L. Matthes, Kaspar Staub

**Affiliations:** 1 Institute of Evolutionary Medicine, University of Zurich, Zurich, Switzerland; 2 Department of History, Ghent University, Ghent, Belgium

**Keywords:** crisis competence, health history, historical epidemiology

## Abstract

Our study aims to enhance future pandemic preparedness by leveraging insights from historical pandemics, focusing on the multidimensional analysis of past outbreaks. In this study, we digitised and analysed for the first time aggregated mortality and morbidity data series from the Russian flu in Switzerland in 1889/1890 and subsequent years to assess its comprehensive impact. The strongest effects were observed in January 1890, showing significant monthly excess mortality from all causes compared to the preceding five years (58.9%, 95% CI 36.6 to 81.0). Even though the whole of Switzerland was affected, the impact varied regionally due to ecological variations. Deaths from other conditions such as tuberculosis and heart disease also increased during this period. A significant drop in birth occurred 9 months later, in the autumn of 1890. Morbidity estimates by physicians suggest that around 60% of the Swiss population fell ill, with regional discrepancies and earlier outbreaks among postal workers (1–2 weeks earlier than the rest of the population). A subsequent spike in all-cause excess and influenza mortality was recorded in January 1894 but more localized than in 1890. Our findings show no cross-protection between the 1890 and 1894 outbreaks.

## Introduction

Prior to COVID-19, the world experienced numerous pandemics caused by viral respiratory infections [1–3]. In the past two centuries, the pandemics of 1957, 1918–20, and 1889–90 were the most severe of the global flu outbreaks [4,5]. Numerous studies have investigated the ‘Spanish flu’ pandemic of 1918–19. By contrast, very little is known about the pandemic that occurred 30 years before, in 1889–90 [6]. The ‘Russian flu’ was the first truly global pandemic outbreak in a world newly connected by rail and covered by mass media [7]. It probably originated in the Russian Empire and spread rapidly along trade routes across Europe within a few weeks from early December 1889. The ‘Russian flu’ pandemic killed about 1 million people (0.07% of the world’s population) [8]. Yet, scholarly attention remains limited. While each pandemic is unique, there are several interrelated factors, for example, in terms of immunity [9], that contribute to their emergence and spread. Therefore, it is crucial to expand our knowledge of the key features of the ‘Russian flu’ [10].

Discussions on the ‘Russian flu’ generally revolve, among other things, around the following unresolved issues: First, the timing: the timeline and the duration are unclear. In today’s perception, the ‘Russian flu’ took place in the winter of 1889–90. However, depending on the country, waves of illnesses and deaths can also be seen in the 3–4 years after 1890. The literature discusses whether the ‘Russian flu’ was a multi-year event, but the available data so far is limited [11–14]. Consequently, historical interpretation of the 1890 flu also requires considering developments in the years before and after the pandemic. This is also important because in multi-wave pandemic events, questions of cross-protection between the waves also become relevant, as has already been shown for the ‘Spanish flu’ [15,16]. To this end, however, it would also be important to identify the causative pathogen genetically, which has not yet been possible for the ‘Russian flu’ [6,12, 17].

Second, the geography: the regional spread is unclear. Scholars have highlighted the role of the railway network in spreading the ‘Russian flu’ across Europe [18,19]. Information from daily newspapers suggests that the pandemic first struck major cities connected by rail, and then spread to other places. Because most existing studies focus on cities, we do not know whether or when not-well-connected places were affected too. There is also a lack of research on how residential altitude and socioeconomic conditions (e.g. the regional level of industrialisation or GDP per capita) impacted pandemic outcomes within countries, despite the demonstrated benefits of historical geographic methods to better understand the spread of the ‘Russian flu’ [20]. Third, there are hardly any studies that compare several pandemic parameters with each other; most of the existing studies on the ‘Russian flu’ focus on either mortality or disease alone. There has also hardly been any broader research to date, although it would be interesting to include other demographic indicators (births, etc. [21–23]) and causes of death in a more holistic view to analyse short and medium-term effects in the year of the ‘Russian flu’ and the years thereafter.

With our study, we contribute to these open questions in the literature by taking Switzerland as a case study and by digitising and analysing in more detail the rich aggregated historical statistical information that has hardly been exploited so far. Switzerland is an interesting case study because at that time - around 1890 - it was still a young nation, small, multi-lingual, internally diverse, regionally and economically heterogeneous, but alpine and still predominantly agricultural, with few urban centres. In the Alps, in particular, some areas were markedly remote and still barely connected. In terms of prosperity, Switzerland was still in the midfield in Europe at the time, as international comparisons of GDP per capita, life expectancy, and average body height show [24]. In addition, the healthcare system in Switzerland has always been organised on a decentralised basis at the level of the 26 member states (cantons), which makes a regional assessment of a pandemic event more relevant.

According to estimates at the end of the 19^th^ century, the majority of the population in Switzerland fell ill with the flu during the ‘Russian flu’ [25]. It has recently been shown that the excess mortality rate in Switzerland, especially in January 1890, was one of the highest in the past 150 years, clearly behind the ‘Spanish flu’ but roughly on a par with the most severe months of the Covid pandemic in autumn 2020 [26]. Despite this historical dimension of the ‘Russian flu’, there are so far only four studies that have included Swiss data on this pandemic [8,19,27,28].

In our study, we will expand the understanding of how and when the ‘Russian flu’ spread in Switzerland and what impact it had by relying on various demographic and health parameters and using newly digitised aggregated historical data. For the years 1890–94, we are investigating the following questions: Which districts in Switzerland were severely affected by flu mortality, and what ecological factors could explain these regional differences? Was there an association between how severely a region or city was hit in 1890 and the years thereafter (cross-protection)? How high was the excess mortality rate in Switzerland and its largest cities? What impact did the pandemic have on related population indicators (births, other causes of death)? Which age groups were particularly affected by the pandemic?

## Materials and methods

For our study, we digitised the following two data sources for Switzerland for the first time and prepared them for analysis.

- A large nationwide survey among physicians on the pandemic (enhanced with standard demographic data): In 1895, Dr. Friedrich Schmid, then the Director of the Swiss Federal Health Office, published the most comprehensive statistical overview ever compiled on a pandemic in Switzerland. This monumental book consists of over 300 pages full of statistics and tables. The core of this book is a large survey by the Swiss Federal Health Office, in which questionnaires were addressed to general practitioners. The questionnaire included questions regarding various pandemic parameters. In the end, over 700 doctors from all over Switzerland responded. The survey has been repeated in the years 1891–94. Also included are many tables on the years before and after 1890, which summarise demographic standard parameters published by the Swiss Federal Statistical Office.
- The weekly bulletin published by the Swiss Federal Health Office with demographic and epidemic core parameters, mostly for the largest Swiss cities. Since the mid-1880s, the Swiss Federal Health Office has published a printed weekly bulletin, which also tabulates the number of various notified infectious diseases, hospitalizations, and deaths. These statistics and standardised tables are weekly and mostly at the level of the 15 largest cities, sometimes also by month at the level of cantons.

Terminology: The sources used in this article refer to both “flu” and “influenza”. Since the causative pathogen of the ‘Russian flu’ has not yet been genetically confirmed (see Discussion), we will use the more inclusive term “flu” rather than “influenza” in this article.

We extracted the following data sets from these two sources:

1. For the 182 districts in Switzerland at this time: Annual deaths due to or involving the flu per 10,000 inhabitants from November 1889 to October 1890, and likewise in the four years thereafter until the end of 1894. These mortality data do not originate directly from the survey of physicians (which was more about morbidity etc.) but were supplemented from the official Swiss death statistics. These numbers included the deaths from/with the flu in aggregate form as deaths from the primary cause of the flu added together with deaths in which the flu was a secondary contributing cause. This extensive mortality table was taken from source a) (see above, Schmid 1895). We examined these data regarding clusters (see below) and at the ecological level of the 180 districts, adding a number of potential explanatory district parameters that could be relevant to our research question: From the same source a) we also took the rough estimates from the physicians surveyed as to what percentage of the population in a district fell ill with the flu between December 1889 and April 1890. From the Swiss area statistics we extracted the altitude above sea level of the district’s main place as well as the cultivable area of each district, the latter to be able to calculate the population density (in people per square kilometre) for each district together with the population data extracted from the 1888 census data. We also extracted from the 1888 census the proportion of people over 60 years of age living in each district, and the proportion of industry and agriculture in the labour force. If a district contained one of the officially 15 largest cities in Switzerland in 1888, then the district was categorised as urban, if not as rural (167 districts). The pandemic of 1890 took place against the background of the industrialisation and urbanisation of Switzerland and the development of new railway networks. In order to see whether a district was well or not well connected in 1890 and 1894, we were able to include the exact number of railway stations per square kilometre as a connectivity proxy for each district from a recently published paper [29]. As a proxy for the economic prosperity of a district, we have used GDP per capita estimates for the year 1888 [30]. And we used the number of hospitals per 1000 inhabitants as an indicator of the medical care supply coverage of a district [31,32].
2. For Switzerland and each of the 15 largest Swiss cities of this era: Monthly number of deaths (all causes) for the years 1885–94 (from sources a and b, see above).
3. For the largest 15 Swiss cities of this era combined: Weekly demographic indicators (live births, stillbirths, infant mortality, and deaths in general and due to respiratory diseases, influenza, tuberculosis, and heart disease) for the period April 1889 until December 1894 (from source b, see above).
4. For the largest 15 Swiss cities of this era combined: Weekly number of all-cause deaths per age group and both sexes for the period January 1890 until December 1894 (from source b, see above).
5. For 6 selected cities for which the data was available: Weekly newly reported flu cases for postal services as compared to the general population for the period January 1890 until March 1890 (from source b, see above).

Information on the population at risk was taken from source b, the Historical Statistics of Switzerland HSSO) [33], and the Swiss Federal census from 1888 [34].

### Statistical methods

Local spatial statistic G was used to cluster the districts with higher or lower excess mortality rates. The G statistic represents z-values. Higher z-values indicate the greater intensity of clustering and the direction (positive (red colour) or negative (blue colour)) indicates a cluster of high or low excess mortality rate. The results for each year are displayed on a choropleth map [35]. Using the ecological determinants for the 182 districts, the association between each determinant and “flu” mortality was examined in an exploratory analysis using robust linear regression (to overcome the issue of outliers and extreme values). The regression coefficients and the 95% confidence intervals are displayed in a figure. Certain associations were also shown as scatterplots with linear regression lines, separated into urban and rural districts.

The monthly mortality for Switzerland, all cities together and for each of the 15 largest cities individually was predicted by using 5 years before the epidemic under the scenario of absence of the epidemic. The monthly number of deaths was modelled using a Poisson distribution, in which the respective population was set as an offset. The monthly seasonality effects were added as monthly cyclicity using sine and cosine functions. The 95% upper and lower prediction intervals were estimated by bootstrapping using 1000 simulations. The respective excess mortalities were calculated by subtracting the expected values from the observed ones. The relative excess mortality rates were then also presented as percentages.

All statistical analyses were performed using R Version 4.3.1. Statistical codes and data are publicly available via GitHub (https://github.com/kasparstaub/RussianfluSwitzerlandhttps://github.com/kasparstaub/RussianfluSwitzerland).

## Results

According to the Schmid survey (source a), around 61% of the population in Switzerland fell ill with the flu between December 1889 and April 1890 (average attack rate). In the first and most severe year, 1889/1890, around 2700 deaths from the flu were recorded. In January 1890 alone, the strongest month, around 3400 more deaths from all causes were recorded than expected (Table 2). In the years 1891 to 1893, there were fewer deaths from the flu, but in the winter of 1893/1894, there were another 2300 deaths from or due to the flu (in January 1894 there were again around 3200 more deaths from all causes than expected). A total of 7200 deaths from and due to the flu were recorded between 1889 and 1894 (corresponding to around 240 deaths per 100,000 inhabitants).

**Figure 1.**
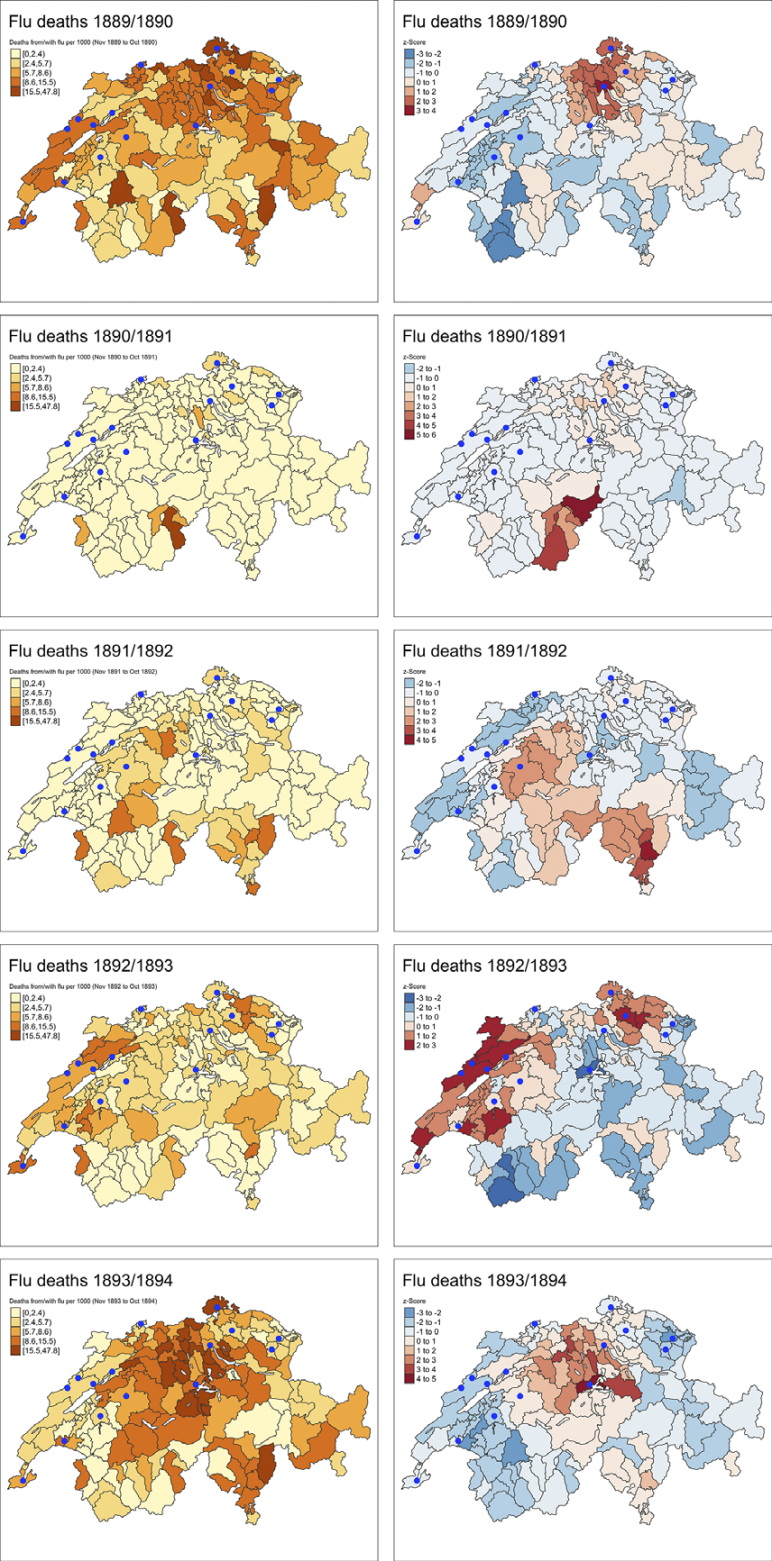
Mortality from/with the flu for 1889/90 and the years thereafter by all districts (left) and results of the local spatial statistic G (right). Higher *z*-values indicate greater intensity of clustering and the direction (positive (red colour) or negative (blue colour)) indicates a cluster of high or low excess mortality rate. Blue dots = The largest Swiss cities. Basis: Source a), which shows the deaths from/with the flu in aggregate form as deaths from the primary cause of the flu added together with deaths in which the flu was a secondary contributing cause.

In the first year of the pandemic (November 1889 to October 1890), the 182 districts in Switzerland were affected to varying degrees in terms of mortality from or contributed to by the flu (range 0.0 to 47.0 flu deaths per 1000 inhabitants, median 7.9) (Figure 1). The cluster analysis revealed a hotspot of increased flu mortality rates in northern Switzerland, in the cantons of Zurich, Aargau, Schaffhausen, and Thurgau. The univariable robust regressions regarding potential ecological explanatory factors at the district level revealed that in this first year of the pandemic, increasing altitude above sea level and a high proportion of agriculture in the labour force were significantly associated with lower flu mortality, while higher population density, more railway stations, a higher proportion of older people over 60 years of age and a higher proportion of industry in the labour force were significantly associated with higher flu mortality (Table 1). In the three following years (1890/91, 1891/92, and 1892/93), flu mortality was then markedly reduced, and only very few districts were more clearly affected, with a flu mortality rate higher than 10 deaths per 1000 inhabitants (median 1890/91 = 0.0, median 1891/92 = 0.9, and median 1892/93 = 2.9). However, even in these weaker flu mortality years, the cluster analysis reveals clear regional hotspots, in different parts of the country in each year (Figure 1).

**Table 1.** The association between ecological determinants and mortality from/with the flu in 1889/90 and 1893/94 for each district as assessed by robust linear regression (to overcome the issue of outliers and extreme values). Significant regression coefficients (*p* < 0.05) are shaded in grey. Basis: Source (a), which shows the deaths from/with the flu in aggregate form as deaths from the primary cause of the flu added together with deaths in which the flu was a secondary contributing cause

	Mortality due/with flu 1889/90				Mortality due/with flu 1893/94			
	Value	SE	t-Value	p	Value	SE	t-Value	p
Altitude (masl)	−0.004	0.002	−2.687	0.009	−0.008	0.002	−4.563	0.000
Population density (pp/km2)	0.000	0.000	2.700	0.006	0.000	0.000	−0.467	0.635
Railway stations per km2 1890	2.900	0.997	2.908	0.003				
Railway stations per km2 1894					−0.377	1.106	−0.341	0.728
GDP p C 1888	0.007	0.004	1.765	0.082	−0.002	0.004	−0.479	0.629
Share people > =60y. 1888	0.554	0.201	2.754	0.007	0.230	0.228	1.007	0.317
Share industry 1888	0.112	0.049	2.271	0.024	0.056	0.056	1.002	0.313
Share agriculture 1888	−0.085	0.040	−2.101	0.037	−0.045	0.046	−0.981	0.324
Hospitals per 1000 pp	−7.365	7.993	−0.922	0.368	−11.804	8.913	−1.324	0.190
Urban vs. rural districts	2.176	1.546	1.408	0.159	−1.127	1.693	−0.665	0.505
Estimated percent sick of population	−0.025	0.033	−0.764	0.452	−0.002	0.038	−0.045	0.964
Mortality due/with flu 1889/90					0.399	0.067	5.935	0.000
Mortality due/with flu 1889–93					0.253	0.041	6.133	0.000

**Table 2.** Modelled monthly all-cause excess mortality (in number of deaths and as a percentage) for December 1889 to February 1890 (left) and the same months in 1893/1894 (right) for Switzerland, all cities together and the individual cities. Significant excess mortality is shaded in orange, significant under-mortality in green. 95% confidence intervals are shown in brackets. Basis: Source (a)

			Winter 1889/1890			Winter 1893/1894		
Place	Population 1890		Dec 1889	Jan 1890	Feb 1890	Dec 1893	Jan 1894	Feb 1894
Switzerland	2,972,024	Deaths (*n*)	5367	9257	5475	5699	9093	5988
		Predicted (*n*)	4826.8 (4576.4 to 5092.7)	5826.8 (5108.2 to 6876.7)	6206.9 (5655.4 to 7372.5)	4914 (4546.4 to 5328.9)	5871.2 (4969.6 to 6938.7)	6253.9 (5467.1 to 7299.9)
		Percent (%)	11.2 (5.8 to 17.2)	58.9 (36.6 to 81)	−11.8 (−23.1 to −2)	16 (8.2 to 25.5)	54.9 (30.2 to 82.3)	−4.3 (−17 to 9.9)
All cities	498,244	Deaths (*n*)	933	1559	906	1075	1401	1274
		Predicted (*n*)	792.2 (724.5 to 884.6)	977.5 (833.3 to 1152.3)	1036.8 (900.6 to 1212.2)	917.4 (840 to 1027.7)	1078.9 (953.8 to 1212.9)	1164 (1061.9 to 1302.1)
		Percent (%)	17.8 (7.1 to 31)	59.5 (35.8 to 87.4)	−12.6 (−24.9 to 1)	17.2 (6 to 28.7)	29.9 (14.2 to 47.9)	9.5 (−2 to 24.4)
Zurich	94,250	Deaths (*n*)	176	296	175	212	323	248
		Predicted (*n*)	133 (110 to 164.2)	168.1 (129.5 to 205.1)	182.7 (149 to 225.2)	177.2 (148 to 207.6)	224.6 (185 to 281.8)	249.2 (214.4 to 294.2)
		Percent (%)	32.3 (10.7 to 66)	76.1 (38.3 to 126)	−4.2 (−23.3 to 22.4)	19.6 (1.9 to 44.2)	43.8 (17 to 77.5)	−0.5 (−16.2 to 19.8)
Geneva	73,661	Deaths (*n*)	124	284	153	166	167	216
		Predicted (*n*)	121.2 (101.4 to 139)	153.8 (116.3 to 198.7)	162.6 (127.9 to 201.6)	148.2 (127.5 to 182.5)	163.8 (137.5 to 198.1)	174.2 (143.5 to 208.1)
		Percent (%)	2.3 (−12.7 to 25.3)	84.7 (40.6 to 144.8)	−5.9 (−25.4 to 21.5)	12 (−6.2 to 33.9)	2 (−14.4 to 21.9)	24 (3.8 to 50)
Basel	72,065	Deaths (*n*)	107	209	98	203	221	165
		Predicted (*n*)	106.2 (84.0 to 129.0)	124.8 (96.0 to 160.0)	131.2 (103.0 to 162.0)	123.9 (93.0 to 158.0)	159.7 (122.0 to 197.0)	163.8 (127 to 195)
		Percent (%)	0.8 (−17.1 to 27.4)	67.4 (30.6 to 117.7)	−25.3 (−39.5 to −4.9)	63.9 (28.5 to 118.3)	38.4 (12.2 to 81.2)	0.8 (−15.8 to 25)
Berne	47,756	Deaths (*n*)	126	165	104	85	134	150
		Predicted (*n*)	97.5 (77.5 to 122.6)	127.7 (102 to 150.5)	131.8 (112 to 161)	99.6 (80.5 to 124.5)	109.5 (89 to 135.6)	124.4 (103.5 to 144.5)
		Percent (%)	29.2 (4.1 to 65.8)	29.2 (7.1 to 68.4)	−21.1 (−35.4 to −1.9)	−14.7 (−30.3 to 7.6)	22.4 (−0.7 to 55.8)	20.6 (0.7 to 50)
Lausanne	34,815	Deaths (*n*)	70	105	61	66	116	97
		Predicted (*n*)	62.1 (45 to 78.1)	73.7 (55 to 93.5)	74.1 (55 to 99)	74.6 (58.5 to 98)	81.8 (57 to 102.5)	85.9 (65 to 105.1)
		Percent (%)	12.6 (−13.6 to 52.3)	42.5 (11.7 to 94.4)	−17.7 (−35.8 to 10.9)	−11.6 (−33.3 to 22.2)	41.9 (7.4 to 96.6)	13 (−11.8 to 54)
St. Gall	29,088	Deaths (*n*)	46	83	52	53	97	56
		Predicted (*n*)	47.8 (33.5 to 60.5)	56 (38.5 to 71)	61.6 (45 to 81)	45.2 (32.5 to 60.5)	58.5 (41 to 75.5)	69 (48 to 92)
		Percent (%)	−3.7 (−27 to 35.3)	48.3 (12.2 to 112.8)	−15.6 (−35.8 to 18.2)	17.4 (−11.7 to 65.6)	65.7 (22.8 to 142.5)	−18.8 (−39.1 to 16.7)
Chaux de Fonds	26,503	Deaths (*n*)	66	56	30	52	59	63
		Predicted (*n*)	36.8 (26 to 48.5)	40.4 (27.5 to 55.5)	41.8 (26 to 59.1)	42.1 (28 to 57.1)	48.8 (33 to 62)	51.6 (33 to 67.5)
		Percent (%)	79.5 (29.3 to 175)	38.8 (0 to 107.6)	−28.2 (−46.4 to 7.1)	23.6 (−8.8 to 79.3)	20.8 (−9.2 to 84.4)	22.2 (−7.4 to 80)
Lucerne	21,110	Deaths (*n*)	29	59	40	34	54	56
		Predicted (*n*)	30.4 (22 to 38.5)	37.7 (24.5 to 50.5)	40.3 (27.5 to 55.6)	34.8 (23.5 to 50.1)	43.1 (28.5 to 56)	44.2 (30 to 58.5)
		Percent (%)	−4.5 (−32.6 to 45)	56.6 (11.3 to 136)	−0.7 (−25.9 to 48.1)	−2.2 (−30.6 to 47.8)	25.2 (−5.3 to 92.9)	26.7 (−5.1 to 80.6)
Neuchatel	16,681	Deaths (*n*)	50	53	35	16	33	35
		Predicted (*n*)	28.1 (19 to 39.5)	32 (23.5 to 43)	33.7 (22 to 46)	25.9 (16 to 37.1)	26.1 (14.5 to 37.5)	28.4 (18.5 to 39.5)
		Percent (%)	77.9 (22 to 194.1)	65.8 (15.2 to 165.3)	3.9 (−23.9 to 66.9)	−38.3 (−56.8 to 0.1)	26.5 (−13.2 to 120)	23.4 (−14.6 to 105.9)
Winterthur	16,423	Deaths (*n*)	20	58	36	33	42	34
		Predicted (*n*)	22.6 (15 to 32.5)	32.5 (18 to 46.1)	36.5 (22 to 50.1)	27.8 (17.5 to 42)	29.8 (18 to 39.5)	34.7 (25.5 to 45.5)
		Percent (%)	−11.7 (−39.4 to 53.8)	78.6 (20.8 to 190)	−1.3 (−29.4 to 56.5)	18.6 (−17.5 to 83.3)	41 (−2.3 to 121.1)	−1.9 (−27.7 to 48)
Biel/Bienne	16,164	Deaths (*n*)	26	45	29	27	42	32
		Predicted (*n*)	20.2 (13 to 32.5)	26 (16 to 38.1)	29.1 (15.5 to 43.5)	27.6 (17 to 43.2)	30.2 (18.5 to 44.6)	32.7 (21 to 45.5)
		Percent (%)	29 (−13.3 to 116.7)	73.3 (15.4 to 181.3)	−0.5 (−31 to 70.6)	−2.3 (−30.8 to 50)	39 (−2.3 to 121.3)	−2.2 (−28.9 to 45.5)
Schaffhausen	12,522	Deaths (*n*)	14	44	12	47	42	25
		Predicted (*n*)	20.2 (12.5 to 32.5)	22.9 (12 to 35.6)	24.2 (13 to 36)	22.1 (13.4 to 36)	29.3 (18 to 43)	28.5 (17.9 to 38.5)
		Percent (%)	−30.8 (−54.8 to 27.3)	92.2 (22.2 to 238.5)	−50.5 (−65.7 to −14.3)	112.4 (30.6 to 292.5)	43.1 (−2.3 to 147.1)	−12.2 (−40.5 to 47.1)
Fribourg	12,382	Deaths (*n*)	26	40	28	31	26	48
		Predicted (*n*)	29.1 (17 to 45.1)	36.6 (22 to 48.5)	38.5 (25.9 to 48.6)	31.2 (22.5 to 44)	31.3 (19.5 to 41.5)	32.7 (21.5 to 48.5)
		Percent (%)	−10.5 (−40.9 to 44.6)	9.4 (−25.9 to 74.1)	−27.3 (−47.2 to 7.7)	−0.7 (−29.5 to 47.6)	−16.9 (−40.9 to 36.8)	47 (2.1 to 128.6)
Locle	11,480	Deaths (*n*)	28	18	29	11	20	17
		Predicted (*n*)	16.9 (11 to 28)	19.3 (11.5 to 31.5)	19.9 (12.5 to 28.5)	16.7 (8.5 to 24)	17 (8 to 28.5)	20.2 (11.5 to 29)
		Percent (%)	65.6 (7.7 to 211.1)	−6.7 (−40 to 63.6)	45.9 (−3.3 to 163.6)	−33.9 (−56 to 22.2)	17.9 (−25.9 to 122.2)	−15.6 (−43.3 to 41.7)

**Figure 2.**
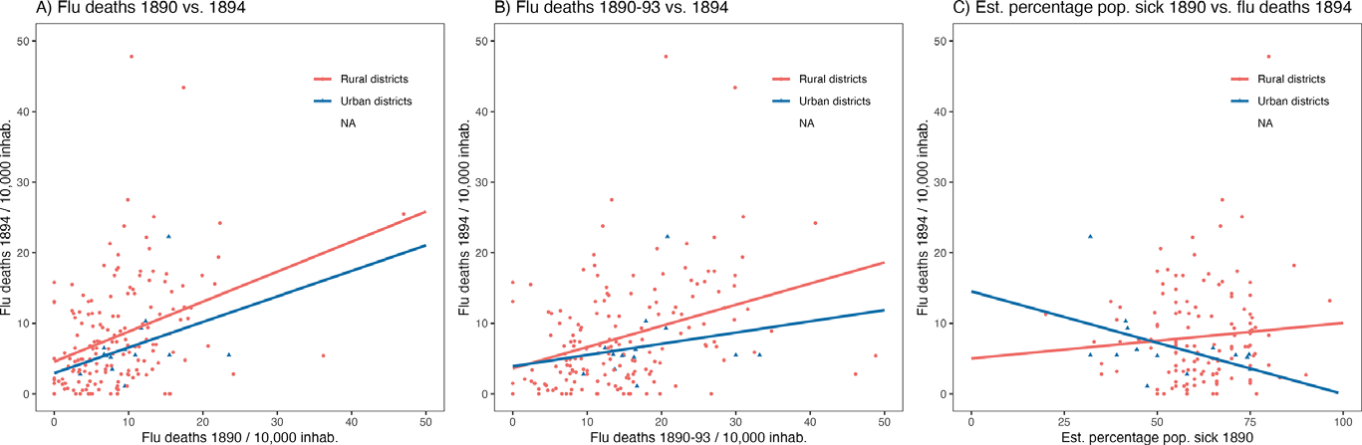
Visualisation of the association (possible cross-protection?) between the district-by-district flu mortality in 1894 (*y*-axis) in comparison with the flu mortality in 1890 (left), 1890–1893 (middle) and with the proportion of the population that fell ill with the flu in 1890 as estimated by physicians. Red = rural districts, blue = districts with the largest cities, lines = linear regression. Basis: Source a), which shows the deaths from/with the flu in aggregate form as deaths from the primary cause of the flu added together with deaths in which the flu was a secondary contributing cause.

In 1893/94, the fifth year after the initial outbreak, there was again a marked increase in flu mortality, again with varying degrees of severity among the districts (range 0.0–47.8 deaths per 1000 inhabitants, median 6.4). This time, the hotspots were in the region of northern Switzerland (around Zurich) and in central Switzerland, and among the ecological explanatory factors at the district level, only a higher altitude above sea level was significantly associated with lower flu mortality (Table 1). To see if there was cross-protection between waves, we associated flu mortality on the district level in the first year of the 1889/1890 pandemic outbreak with flu mortality in the winter of 1893/1894. The robust regressions (Table 1) show that districts that had a higher flu mortality in the first year of the pandemic also had a significantly higher flu mortality in the winter of 1893/1894. This result also holds if we sum up the flu mortality of the years 1889 to 1893 to a cumulative flu mortality in the years before the winter of 1893/1894. This positive association, which speaks against cross-protection in the case of flu mortality, is also visualised in Figure 2 (A & B). The association between the percentage of the population per district estimated by the physicians to have fallen ill in the wave at the beginning of 1890 and mortality from/with flu in 1894 was flat overall (Table 1). However, there was a negative association in the largest cities (Figure 2C): The higher the percentage of ill persons in 1890, the lower the mortality in 1894. Overall, however, the indications of cross-protection were also rather weak for morbidity in 1890.

**Figure 3.**
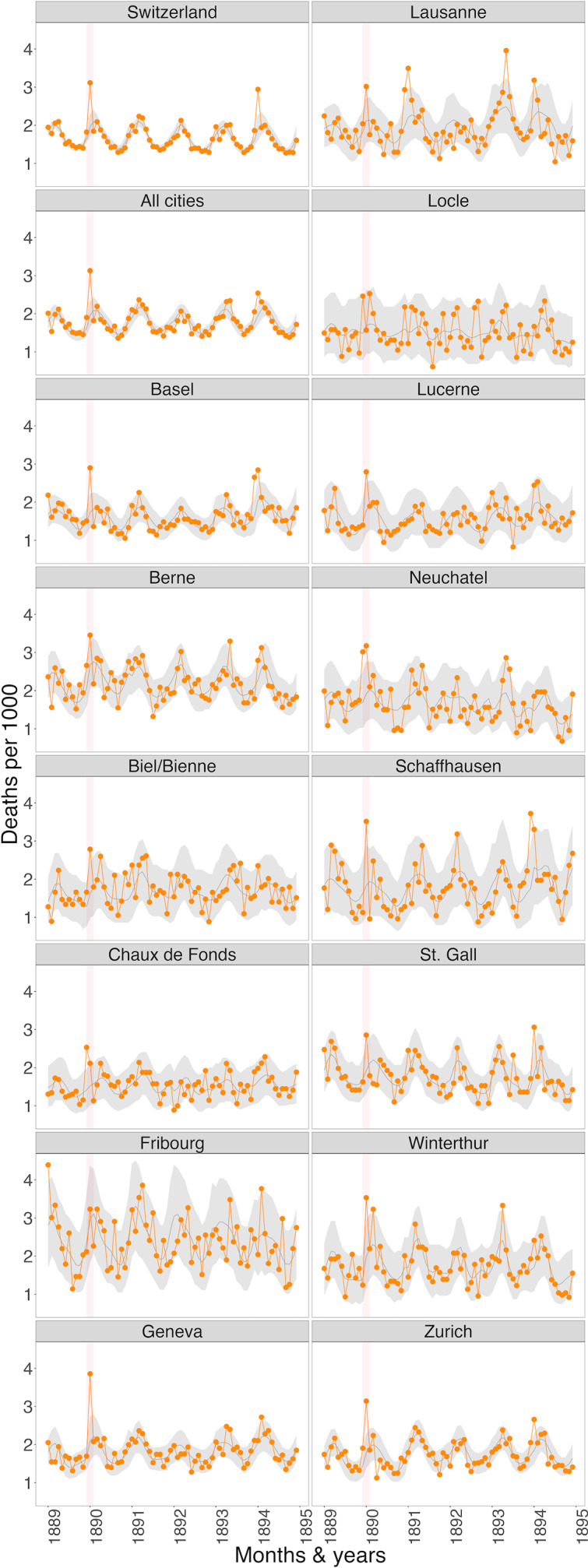
Modelled monthly all-cause excess mortality (in deaths per 1000 population) for January 1889 to December 1894 for Switzerland, all cities together and the individual cities. Orange line/dots = observed deaths, dark grey line = modelled expected deaths, light grey area = 95%CI area of the expected values, red shaded bar = January 1890 (main peak). Basis: Sources (a) and (b).

The monthly excess mortality by all causes of death for Switzerland, all cities together, and for the individual cities is shown in Figure 3. In the winter of 1889/1890, the month with the highest excess mortality was January 1890: for Switzerland as a whole, the excess mortality then amounted to 58.9% (95%CI 36.6 to 81.0), for all cities together 59.5% (95%CI 35.8 to 87.4) (Table 2). Of the 14 largest cities, 12 had significant excess mortality in January 1890, with the most affected were Schaffhausen with 92.2% (95%CI 22.2 to 238.5), Geneva with 84.7% (95%CI 40.6 to 144.8), Winterthur with 78.6% (95%CI 20.8 to 190.0), Zurich with 76.1% (95%CI 38.3 to 126.0), and Biel with 73.3% (95%CI 15.4 to 181.3). Locle and Fribourg recorded no excess mortality. There is no discernible regional pattern or influence of city size. 5 of the 14 largest cities also had significant excess mortality in December 1889; for the Jura cities of Chaux-de-Fonds, Neuchâtel, and Locle, this was even higher than in January 1890. In February 1890, Switzerland as a whole and many cities recorded under-mortality: for Switzerland this amounted to −11.8% (95%CI −23.1 to −2.0), with particularly high under-mortality in Basel −25.3% (95%CI −39.5 to −4.9), Berne −21.1% (95%CI −35.4 to −1.9) and Schaffhausen −50.5% (95%CI −66.7 to −7.7). In the winter of 1893/1894, there was another significant excess mortality, which in January 1894 was again 54.9% (95%CI 30.2–82.3) for the whole of Switzerland and thus almost as high as in January 1890, although this time the cities were slightly less affected at 29.9% (95%CI 14.2–47.9) than in January 1890.

**Figure 4.**
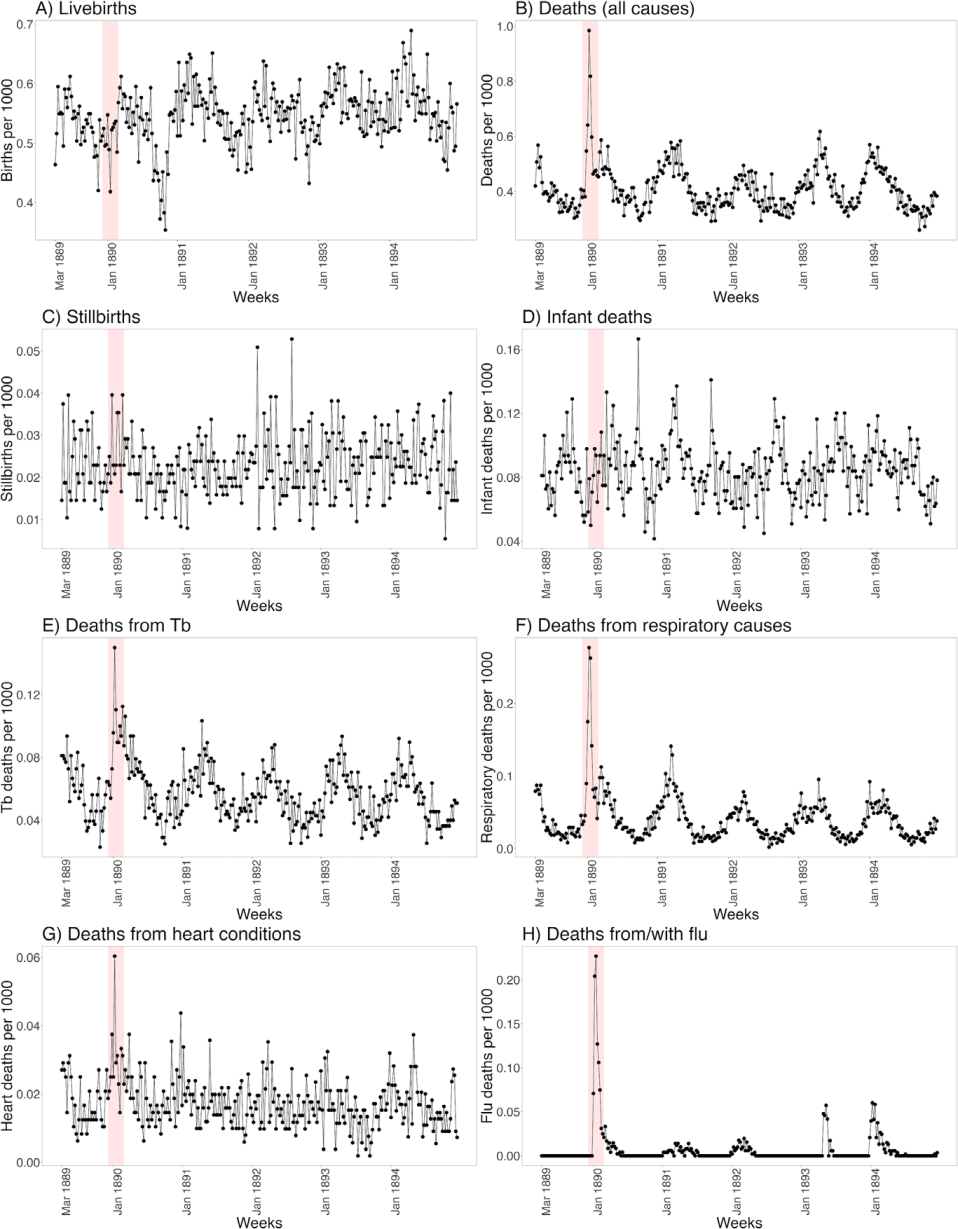
Visualisation of various weekly demographic indicators for all the largest Swiss cities combined. Red shaded bar = January 1890 (main peak). Basis: Sources b).

For all 14 largest cities together, certain demographic parameters were even shown per reporting week for the period from March 1889 (Figure 4). In the case of live births (A), a clear reduction in the number of live births can be seen for several weeks in the fall of 1890, around 9 months after the peak of the pandemic in January 1890. For all deaths (B), it can be seen that there were around 5 weeks in January 1890 in which there was increased mortality (January 1894 is barely visible in the cities from a weekly perspective). In the case of stillbirths (C), slightly higher values can be seen in January 1890, and in the case of infant deaths (D) in the months after January 1890. However, these values cannot be modelled because the period before March 1889 was not recorded. In addition to flu/influenza as a cause of death (H), the causes of death TB (E), respiratory diseases in general (F), and heart conditions (G) also show a peak during the weeks around January 1890.

**Figure 5.**
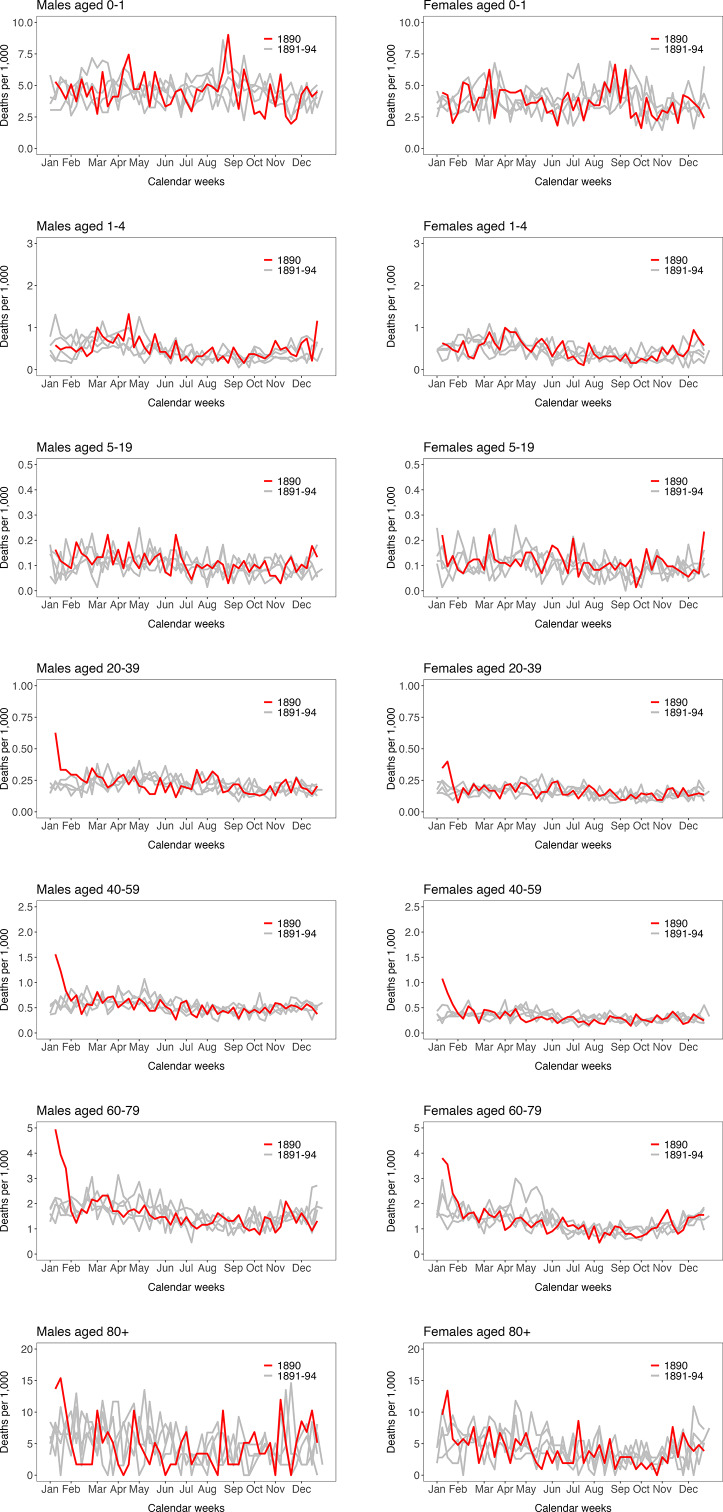
Weekly deaths (all causes) for males (left) and females (right) per 1000 people by age group for the largest Swiss cities together. Red = the year 1890, grey = the years 1891–1894. Basis: Source b).

Again, for all 14 largest cities together, the weekly number of deaths per age group and sex for the year 1890 (red line) can be compared with the years 1891 to 1894 (grey lines) (Figure 5). The different *y*-axes show the different levels of mortality in the various age groups (mortality was lowest in the age groups 5–19 and 20–39 years). It can also be seen that for both sexes, mortality for the weeks in January 1890 (red) from the age group 20–39 and older are clearly above those for the years 1891 to 1894 (grey lines).

**Figure 6.**
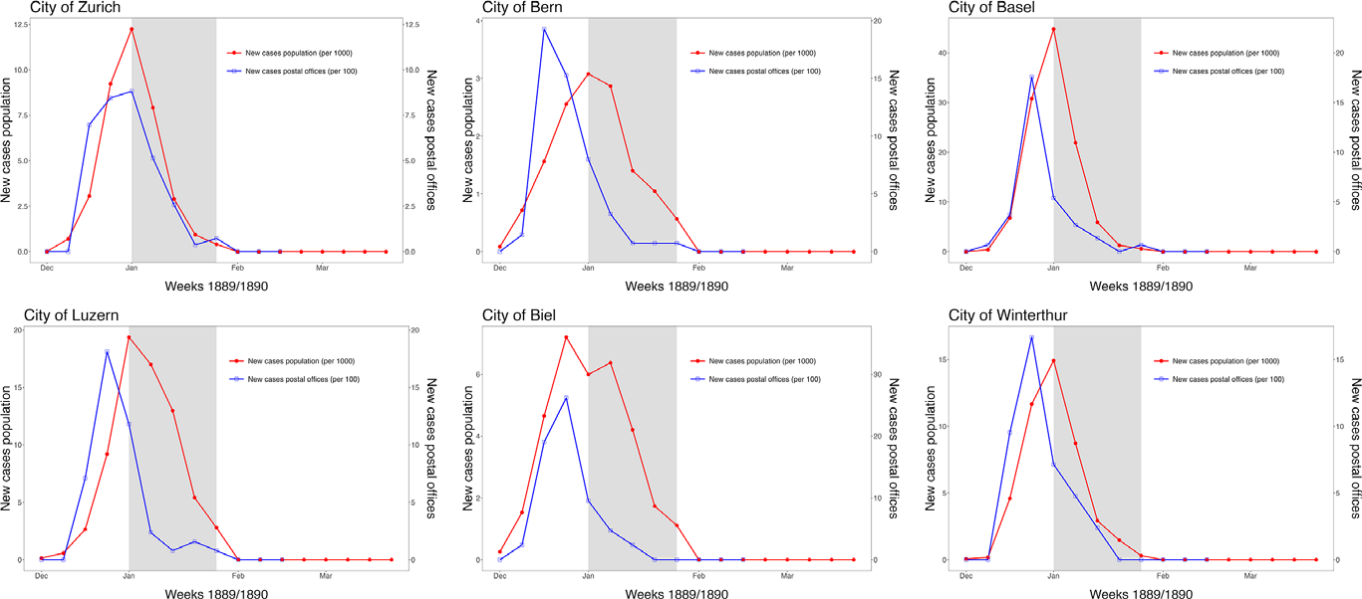
Weekly new illnesses in the population (red) and in local post offices (blue) in six selected Swiss cities. Shaded grey = month with the highest mortality in the respective cities. Basis: Source (a). These incidence data were only recorded for 1890.

For 6 selected cities (Zurich, Lucerne, Bern, Basel, Biel, Winterthur), the weekly reported new illnesses in the population (there was certainly underreporting because there was no mandatory reporting of influenza before 1918) can be compared with the weekly number of employees absent due to illness in local post offices for the period December 1889 to March 1890 (Figure 6). The epidemic waves show good synchrony in timing and a duration of around 6–8 weeks. In the post offices, where the employees were supposedly exposed more and earlier than the general population due to increased customer traffic, the wave usually rose around 1–2 weeks earlier than in the population as a whole. The month with the highest flu mortality in the respective cities is shaded grey. A further temporal shift can be seen here, with mortality presumably occurring a few weeks later than morbidity.

## Discussion

For the present study, we digitised and analysed various aggregated mortality and morbidity data series for the first time to obtain a more comprehensive picture of the influence of the Russian flu in Switzerland in 1889/1890 and the years that followed. We see that the impact was multifaceted, varied regionally, and was both immediate and intermediate. The immediate impact of the Russian flu in Switzerland was strongest in January 1890, at least in terms of mortality. Even though the whole of Switzerland was affected, there were regional differences, which can be partially explained by various ecological factors. In January 1890, there was a considerable excess mortality rate, and other certified causes of death such as tuberculosis or heart conditions were also higher. In addition, 9 months later, in autumn of 1890, far fewer births were observed (missing births). In terms of morbidity, physicians estimated that a total of 60% of the population had fallen ill between December 1889 and April 1890, although this again varied from region to region, with particularly exposed post office employees at transport centres, for example, falling ill 1–2 weeks earlier than the rest of the population in the same towns and villages. Regarding the following years, only in January 1894 was there another increase in all-cause excess and flu mortality, but more locally limited than in 1890. In terms of mortality, we did not find cross-protection between 1890 and 1894.

In our study, we analysed the spatial associations between contextual factors and annual flu mortality at the district level. We find that for the first pandemic year, 1889/1890 a poorer integration into the railway network, a lower population density, a lower proportion of people over 60 years of age, and a lower proportion of industrial labour force structure were associated with lower flu mortality. The fact that the virus had spread regionally along the railway routes in Switzerland in 1889/1890 had already been shown earlier [19]. We show for the “Russian flu” of 1890, what we have already shown for the “Spanish flu” of 1918–20, that the incidence and mortality from influenza is lower in the same topographically higher-altitude residential areas [35,36]. How this possibly protective effect of altitude can be explained remains to be clarified. In addition to factors of remoteness or socio-demographic structure, less pollution, more sunshine (and thus higher vitamin D levels) or other environmental factors could also come into question. We also show that the impact of the “Russian flu” in Switzerland was regionally heterogeneous among districts and cities. This emphasises the importance of studying the health impact of pandemics on a society regionally to better understand the pattern.

Following the monumental statistical work by Schmid published in 1895 [25], our study is the most comprehensive statistical analysis of the “Russian flu” pandemic in Switzerland to date (previous works have only analysed individual aspects) [19,37]. In terms of temporal patterns, Switzerland shows a somewhat particular pattern: after the major outbreak in January 1890, it was not until the winter of 1893/1894 that there was another increase in mortality, both in general and due to the flu, and this varied geographically. In other countries, regions, and cities, increased mortality rates were already seen again in the years immediately following 1890 [7, 13,14,38]. Therefore, in the limited existing literature, the “Russian flu” is considered to be multi-wave. For Switzerland, it is not possible to provide a clear answer to the question of multiple waves, as there were four winters with rather low flu activity between the waves. Regarding cross-protection between the 1890 and 1894 waves, we see no clear regional pattern for the Swiss districts and mortality, which could also be due to the temporal distance or the possibly insufficient precision of the available and analysed parameters. From the few existing studies, a so-called J-pattern has been proposed regarding the age pattern in the mortality of Russian flu (slightly increased mortality in young children, and then a steadily increasing mortality from about 20 years of age) [7,27,38]. Increasing age as a risk factor also applies to Switzerland, but at least in Swiss cities, we do not see increased mortality among younger children in 1890 compared to the years thereafter (unfortunately the data in the years before 1890 are not available). However, this could also be related to different denominators (in our case the population in the respective age groups) or different aggregation levels (in our case weeks).

We confirm other studies that in 1890 there was an interaction in mortality with also increased tuberculosis deaths (either as a risk factor in the sense of pre-existing diseases, or also in the sense of misdiagnosis in the causes of death) [37], but we also show that heart disease as a cause of death shows a peak in January 1890. How this association is to be explained (a direct or indirect consequence of the many infections?) cannot be answered with our aggregated data; here, studies would have to follow based on hospital records. Causes of death other than flu or pneumonia should therefore also be considered if the mortality impact of the Russian flu is to be described more comprehensively.

The “Russian flu” in 1889/90 was one of the three strongest pandemics in Switzerland’s history since the end of the 19th century in terms of mortality. Depending on whether annual or monthly excess mortality is calculated for all causes of death, the “Russian flu” is the second or (after Covid-19) third strongest pandemic, but both are well behind the “Spanish flu” of 1918–20 [26]. It is in the nature of pandemics triggered by a respiratory virus that the majority of people who fall ill survive the infection and/or illness. The overall health impact of a pandemic therefore goes beyond mortality and affects a wide range of factors such as incidences, absences from work, or even an incomplete or prolonged recovery (post-viral symptoms) [39–41]. Contemporary experts shortly after the “Russian flu” characterised the pandemic as the “enormous influenza epidemic”. This is illustrated in particular by the fact that, on average, almost two-thirds of the Swiss population were ill [25]. At the time, Otto Nägeli was a physician in the municipality of Ermatingen in eastern Switzerland. In February 1890, shortly after the strong wave in January 1890, he visited every house in the municipality and made an enquiry as to who lived in the houses and who – according to symptoms – was suffering from the flu [42]. His enquiry covered a total of 295 families and 1330 people. Nägeli was able to show that there were 813 illnesses in the municipality (61%). Around 19% of the cases were severe, and there were around 6% reinfections. In the municipality, women were more likely to be infected than men (65% vs. 57%), and in general, the infection rates were lower in younger and older people than in people between 5 and 60 years of age.

With regard to the sharp reduction in births 9 months after the peak of the pandemic, we also find similar patterns in other pandemics. Birth rates are known to respond to pandemics and other crises [43], including heatwaves and natural disasters such as tsunamis [44,45]. In the case of pandemics and epidemics, it has been shown that birth rates appear to drop approximately 9 months after the peak of an outbreak (as was the case with SARS-CoV-1, Zika, and to a lesser extent Ebola), followed by a rebound in births [46]. The reasons for this pattern are multifactorial but are likely to be related to deliberate postponement of conception, as well as illness-related fertility restrictions and natural abortions early in pregnancy during the peak of the outbreak. Most of the evidence on historical pandemics comes from research on the 1918–20 influenza pandemic (“Spanish flu”), when births declined 9 months after the pandemic peak in Scandinavia [21,23,46], Britain [47–49], Japan [48], and the United States [21,49]. However, some of these aspects are currently being debated in the literature, for example, whether the 1918–20 pandemic or the end of the First World War is more likely to be associated with these changes in birthrates [22,50].

The pathogen causing the “Russian flu” pandemic remains unknown. Due to the lack of human samples, genetic detection of the pathogen of the ‘Russian flu’ has been unsuccessful so far. For decades, it was assumed that it was an influenza A virus (H2N2 or H3N8, which is no longer found in humans) [6,17,51]. Considering the observed periodicity of the waves in 1890 and the following years, it would fit relatively well with the periodicity of the influenza virus [52]. More recently, also in the wake of COVID-19, it has been suggested that it was a coronavirus (OC43?). This theory is indirectly supported by temporal correlations of the common ancestors of today’s coronaviruses dating back to ca. 1890 [52,53] and by specific symptoms reported by physicians, such as the loss of smell and taste [54,55]. Some however voice caution as there is little hard data (the symptoms could pertain to another disease) and there is no genetic material from that era to verify yet [6,11].

Our study has several limitations. Firstly, although our study is based on newly digitised archive data analysed for the first time, this is aggregated information published by the authorities and not individual data, which would allow an even more precise assessment of the effect of age, sex, or socio-economic background. However, the quality of the official data at the time was very good in the case of the standard vital statistics and also in the case of the large survey of physicians conducted by the federal health authorities. Secondly, there is relatively abundant information on mortality during the “Russian flu”, but the evidence base on morbidity is much less comprehensive. If more information on illnesses, absences from work, or hospitalisations were accessible, the overall burden of disease could be better reconstructed. Thirdly, we have reconstructed the demographic and health impact and epidemiological course of the Russian flu in this study, but we have not investigated how the authorities reacted to the outbreak with measures or how the population perceived this pandemic. Certainly, no far-reaching and comprehensive measures were taken by the authorities, which our extensive archive research would have uncovered. Nevertheless, these important layers would be part of a comprehensive reconstruction of the pandemic and should be analysed in follow-up studies. Fourthly, our study is limited by the availability of administrative indicators and their temporal coverage. This also means that we can only describe and show associations, but not establish causal links.

## Conclusion

Knowing the specific characteristics of past pandemics, in the sense of scenarios from the past, can help to better assess current and future challenges. However, there is still too little information on the “Russian flu” [56], one of the strongest pandemics of the last 150 years after the “Spanish flu” of 1918–20, despite the excellent sources and data available. Using Switzerland as an example, our study aims to contribute here and indicate that the impact of this pandemic on disease and mortality in Switzerland was substantial. However, our study also shows that this influence was not only immediate but was rather heterogeneous in terms of region. Future studies should therefore collect and analyse individual data so that an even more diversified approach is possible.

## Data Availability

https://github.com/kasparstaub/RussianfluSwitzerland

## Statistical code

https://github.com/kasparstaub/RussianfluSwitzerland
